# Outer membrane-anchoring enables LpoB to regulate peptidoglycan synthesis rate

**DOI:** 10.1016/j.tcsw.2022.100086

**Published:** 2022-10-20

**Authors:** Ali A. Kermani, Jacob Biboy, Daniela Vollmer, Waldemar Vollmer

**Affiliations:** Centre for Bacterial Cell Biology, Biosciences Institute, Newcastle University, Richardson Road, Newcastle Upon Tyne NE2 4AX, UK

**Keywords:** Peptidoglycan, Outer membrane lipoproteins, mis-localized LpoB, Penicillin-binding protein, Osmotic upshift

## Abstract

Peptidoglycan (PG) is an essential component of the cell envelope in most bacteria, responsible for maintaining the shape of the cell and protecting the cell from environmental stresses. The growth of the PG layer during cell elongation and division is facilitated by the coordinated activities of PG synthases and hydrolases. PG synthases are regulated from inside the cell by components of the elongasome and divisome complexes driven by the cytoskeletal proteins MreB and FtsZ. In *Escherichia coli* the PG synthases PBP1A and PBP1B require the activation by outer membrane (OM)-anchored lipoproteins LpoA and LpoB, respectively. These have an elongated structure and are capable to span the periplasm to reach their cognate, cytoplasmic membrane (CM)-anchored PG synthase through the PG layer. Presumably, the Lpo proteins activate the PBPs at sites where the PG mesh is stretched or defective, resulting in coupling of PG synthase activation with cell growth or PG repair. Here we investigated the importance of OM-anchoring on the function of Lpo proteins in regulating PG synthesis in response to environmental stresses. We investigated the effects of an artificially CM-tethered LpoB on cell morphology and PG synthesis. Our results indicate that mis-localization of LpoB affects the growth and morphology of cells in high osmolarity growth medium, and PG synthesis rate upon an osmotic upshift.

## Highlights

• Mis-localized LpoB is able to activate PBP1B.
• LpoB mis-localization affects cell growth upon osmotic upshift.
• LpoB mis-localization alters the cell morphology upon osmotic upshift.
• Peptidoglycan composition is not significantly affected by mis-localization of LpoB.
• Mis-localization of LpoB affects PG synthesis rate after an osmotic upshift.

## Introduction

The peptidoglycan (PG) layer, called sacculus, surrounds the bacterial cytoplasmic membrane (CM) and is required to maintaining the shape of the cell and protecting the cell from bursting due to the turgor (Vollmer et al., 2008). PG forms a mainly single-layered, mesh-like macromolecule in *Escherichia coli* and other Gram-negative bacteria, and is multilayered in Gram-positive bacteria such as *Bacillus subtilis*. PG is composed of glycan chains made of alternating *N*-acetylglucosamine (Glc*N*Ac) and *N*-acetylmuramic acid (Mur*N*Ac) residues that are connected by short peptides (Vollmer and Bertsche, 2008).

The precursor for PG synthesis, lipid II, is synthesized at the cytoplasmic side of the CM and transported to the periplasmic side, where it is utilized by glycosyltransferases (GTases) to polymerize the glycan chains and dd-transpeptidases (TPases) that form the peptide cross-links (Typas et al., 2012, Egan et al., 2020). PG synthesis is mediated by distinct classes of PG synthases. Class A penicillin-binding proteins (PBPs) are bifunctional enzymes with GTase and dd-TPase activities. Class B PBPs are dd-TPases that work in complex with the SEDS family of GTases (Meeske et al., 2016, Taguchi et al., 2019) and class A PBPs (Banzhaf et al., 2012, Bertsche et al., 2006). PBP1A and PBP1B belong to class A PBPs and play a main role in PG synthesis in *E. coli* and the presence of at least one of them is essential for survival (Egan et al., 2020, Yousif et al., 1985). PBP2 and PBP3 are class B PBPs with essential roles in cell elongation and division, respectively (Sauvage et al., 2008).

Growth of the PG layer during the elongation of a rod-shaped cell and cell division requires the coordination between the synthesis of new PG and its incorporation into the sacculus by PG synthases and the cleavage in the sacculus and removal of old material by PG hydrolases (Typas et al., 2012, Egan et al., 2020). Several PG hydrolases have been shown to be regulated by outer membrane (OM) anchored lipoproteins (Uehara et al., 2010, Banzhaf et al., 2020, Gurnani Serrano et al., 2021, Mueller et al., 2021). PG synthases are integral CM proteins and regulated from inside the cell by proteins associated with the dynamic elongasome and divisome complexes driven by the cytoskeletal proteins MreB and FtsZ, respectively (Typas et al., 2012, Egan et al., 2020). However, PBP1A and PBP1B of *E. coli* each require a cognate OM-anchored lipoprotein, LpoA and LpoB, respectively, in order to be functional in the cell (Typas et al., 2010, Paradis-Bleau et al., 2010). *Pseudomonas aeruginosa* lacks LpoB and the function of PBP1B is dependent on LpoP (Greene et al., 2018). Lpo proteins bind to and activate the PBPs: LpoA activates the TPase (Typas et al., 2010, Egan et al., 2014, Lupoli et al., 2014) and GTase (Sardis et al., 2021) of PBP1A, LpoB activates both activities of PBP1B (Typas et al., 2010, Egan et al., 2014, Mueller et al., 2019, Pazos et al., 2018, Egan et al., 2018, Catherwood et al., 2020) and the activation of the TPase domain of PBP1B is modulated by CpoB (Gray et al., 2015). LpoP stimulates both activities of PBP1B from *P. aeruginosa* (Caveney et al., 2020).

*E. coli* LpoB is required for the activity of PBP1B when the enzyme is reconstituted in a membrane (Hernández-Rocamora et al., 2018) which presumably explains the strict requirement of LpoB for PBP1B function in the cell. *E. coli* requires either PBP1A/LpoA or PBP1B/LpoB to maintain viability, and PBP1B/LpoB has a more prominent role in cell division whereas PBP1A/LpoA appears to be more important during cell elongation (Bertsche et al., 2006, Banzhaf et al., 2020, Pazos and Vollmer, 2021). PBP1B/LpoB, but not PBP1A/LpoA, have an additional role in stabilizing the PG under certain OM stress conditions (Morè et al., 2019). PBP1B/LpoB are needed to prevent lysis when the biogenesis of lipopolysaccharide (LPS) or its export to the OM is impaired, presumably because this OM stress causes defects in the PG layer that need to be repaired. This PG repair pathway requires the GTase function of PBP1B, LpoB and the dd-carboxypeptidase PBP6 (Morè et al., 2019). PBP1B/LpoB are needed for the survival of cells exposed to certain antibiotics (García del Portillo and de Pedro, 1990) or with impaired cell division (de Pedro et al., 2001), suggesting that PG repair is needed under these conditions.

The structures of LpoA and LpoB determined by NMR spectroscopy and/or X-ray crystallography have shed more light on the regulatory roles of these lipoproteins (Egan et al., 2014, Jean et al., 2014). Although, LpoA and LpoB adopt distinct folds in their structures, overall, both proteins have elongated molecular shapes. LpoA is composed of two distinct domains (Jean et al., 2014). The *N*-terminal domain of LpoA is comprised of α-helices that form a helix-turn-helix tetratricopeptide-repeat (TPR)-like motif. The authors modelled the full-length LpoA based on the structure of the homologous C-terminal domain of LpoA from *Haemophilus influenza*, and the radius of gyration determined by small angle X-ray scattering (Jean et al., 2014). Subsequently, the Saper group determined the crystal structure of full-length LpoA from *H. influenza* (Sathiyamoorthy et al., 2017) and the *N*-terminal domain of *E. coli* LpoA (Kelley et al., 2019).

LpoB has an elongated unstructured *N*-terminus of 54 amino acids attached to a globular C-terminal domain (Egan et al., 2014). LpoB binds to the small noncatalytic UB2H domain of PBP1B, situated between its GTase and TPase domains (Egan et al., 2014). This binding is essential for both GTase and TPase activities of PBP1B and mutating residues involved in the interaction surface between LpoB and PBP1B impairs the activation of PBP1B in vitro and its function in the cell. It is intriguing that the OM-anchored LpoB uses its elongated structure, ∼145 Å long, to span the periplasm and reach PBP1B’s UB2H domain through the PG layer. It has been proposed that Lpo proteins activate their cognate PBPs at sites where PG mesh is stretched, allowing the coupling of PG synthase activation with cell growth (Typas et al., 2012). In addition, LpoB might activate PBP1B at defective sites in the sacculus that require PG repair (Morè et al., 2019). However, it has not been formally proven that the OM anchoring of Lpo proteins regulates PG synthesis in response to the properties of the PG. In this study, we made use of the finding that a CM-anchored LpoB can support growth of *E. coli* cells in the absence of PBP1A (Typas et al., 2010). We compared phenotypes, cell morphology and PG synthesis in cells lacking PBP1A and having CM- or OM-anchored LpoB. Our data support the model that the OM-anchoring of LpoB regulates PG synthesis.

## Results

**Fig. 1:**
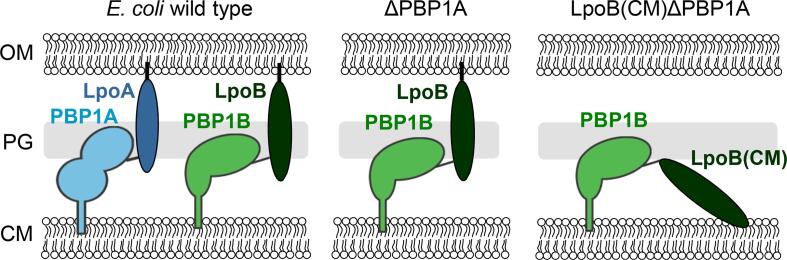
Schematic representation of mis-localization of LpoB. In wild type *E. coli* the OM-anchored lipoproteins LpoA and LpoB activate their cognate major PG synthases PBP1A or PBP1B, respectively, through the PG layer. The cell needs either PBP1A/LpoA or PBP1B/LpoB for growth and the absence of both is synthetic lethal. ΔPBP1A cells have PBP1B as the only major class A PBP PG synthase. In LpoB(CM) ΔPBP1A, the mis-localized LpoB remains tethered to the CM.

CM-anchored LpoB is able to support cell growth in the absence of PBP1A, albeit cells were sick at high osmolality, suggesting that this version of LpoB is at least partially functional (Typas et al., 2010). In this work we aimed to decipher the cellular consequences of the mis-localized LpoB, for PG synthesis. We performed all experiments in strains lacking PBP1A, rendering LpoB and PBP1B essential, in which LpoB was either correctly anchored to the OM (ΔPBP1A) or mis-placed by anchoring to the CM [LpoB(CM) ΔPBP1A] (Fig. 1).

### LpoB mis-localization affects cell growth upon osmotic upshift

**Fig. 2:**
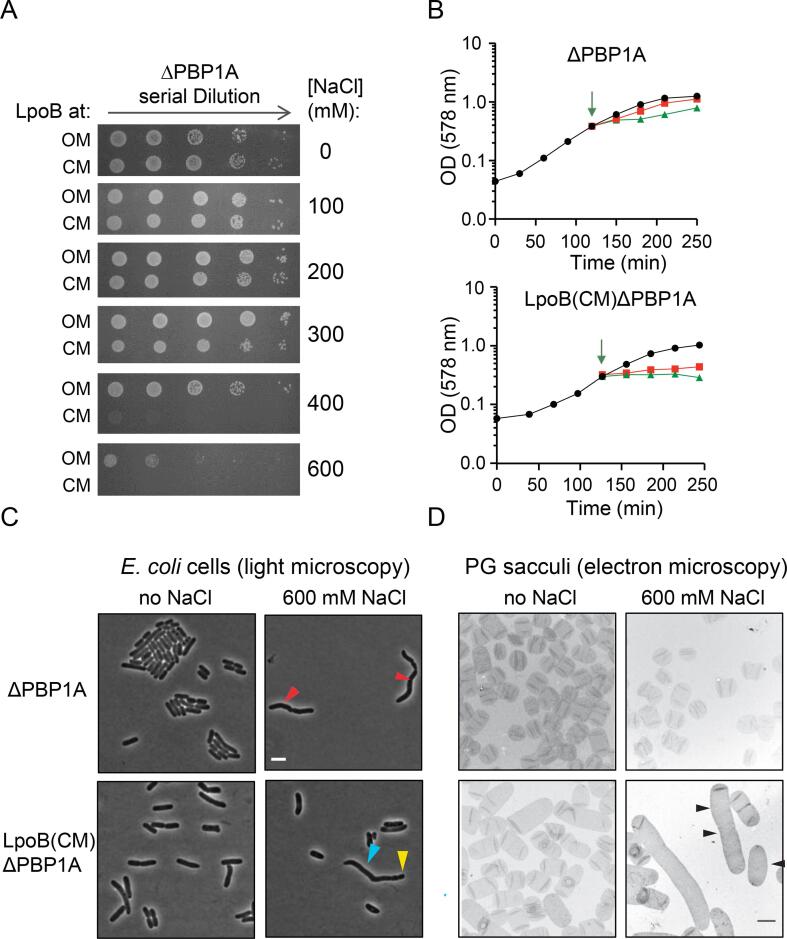
LpoB mis-localization impairs growth and cell morphology. (**A**) Growth defect of LpoB(CM) ΔPBP1A cells at high osmolarity. ΔPBP1A and mis-localized LpoB(CM) ΔPBP1A cells were serially diluted and spotted on LB agar plates supplemented with 0 to 600 mM NaCl. (**B**) Growth curves of ΔPBP1A and LpoB(CM) ΔPBP1A cells. Cells were grown in LB media without NaCl (black circles) until the log phase. The culture was divided into three parts which received no, 400 mM (red square) or 600 mM (green triangle) NaCl. The arrow indicates the time at which NaCl was supplemented to the growth media. (**C**) Phase contrast microscopy of cells and (**D**) EM of purified sacculi from ΔPBP1A and mis-localized LpoB(CM) ΔPBP1A cells with or without an upshift of NaCl. In (**C**) red arrow heads indicate longer size and irregularity in ΔPBP1A cells; blue and yellow arrow heads point to irregularities and formation of smaller cells, respectively, under high osmolarity conditions in LpoB(CM) ΔPBP1A cells. In (**D**) black arrow heads indicate abnormalities in mis-localized LpoB(CM) ΔPBP1A cells. Bar represents 2 μm. (For interpretation of the references to colour in this figure legend, the reader is referred to the web version of this article.)

In order to examine the effects of CM localization of LpoB on bacterial growth and survival under different osmotic conditions, isogenic ΔPBP1A and LpoB(CM) ΔPBP1A mutants were spread on LB-agar plates supplemented with 0 to 600 mM NaCl. LpoB(CM) ΔPBP1A cells grew poorly at high salt concentration (400 mM) and failed to form any visible colonies at 600 mM NaCl (Fig. 2A), confirming previous observations (Typas et al., 2010). In contrast, ΔPBP1A cells with correctly OM anchored LpoB grew at 400 mM NaCl and showed reduced growth only at 600 mM NaCl. To further investigate these effects, we cultured both strains in LB media and monitored their optical density (OD) during the experiment. Both strains grew similarly until they reached exponential phase of growth. At this time the cultures were divided into three aliquots of which two were given an osmotic shock by adding 400 or 600 mM NaCl into growing cultures (Fig. 2B). One aliquot served as a control and received no NaCl. Although the ΔPBP1A strain initially reduced its growth at 600 mM NaCl it was able to resume growth over the time course of experiment. By contrast, the OD of the LpoB(CM) ΔPBP1A cultures with 400 or 600 mM NaCl remained steady over time indicating a halt in cell growth (Fig. 2B). These results show that the outer membrane-anchoring of LpoB protects cells from osmotic upshift, likely by regulating the PG biosynthesis rate, and that artificially CM-anchored LpoB fails to support growth at higher osmolality.

### LpoB mis-localization alters the cell morphology upon osmotic upshift

To better understand the effects of mis-localization of LpoB into the CM on cell growth, ΔPBP1A and LpoB(CM) ΔPBP1A cells transferred into media with or without 600 mM NaCl were imaged by phase contrast microscopy. ΔPBP1A cells grown without salt were largely homogeneous in size but cells often became elongated and bent after an osmotic upshift caused by 600 mM NaCl (Fig. 2C). LpoB (CM) ΔPBP1A cells appeared wider and longer compared to ΔPBP1A cells when grown at no salt condition. However, these cells became larger and had irregular cell shape upon osmotic upshift, compared to the ΔPBP1A cells (Fig. 2C).

We also isolated the PG sacculi from the cells and visualized them by electron microscopy (Fig. 2D). While the sacculus isolated from both strains grown without salt had similar short rod-shape, LpoB(CM) ΔPBP1A sacculi isolated from osmotically shocked cells were often elongated and had somewhat irregular diameter at different regions of the lateral wall of the sacculi, suggesting that LpoB(CM) ΔPBP1A cells might mis-regulate sacculus growth upon osmotic shock.

### Mis-localization of LpoB has small effect on PG composition

**Fig. 3:**
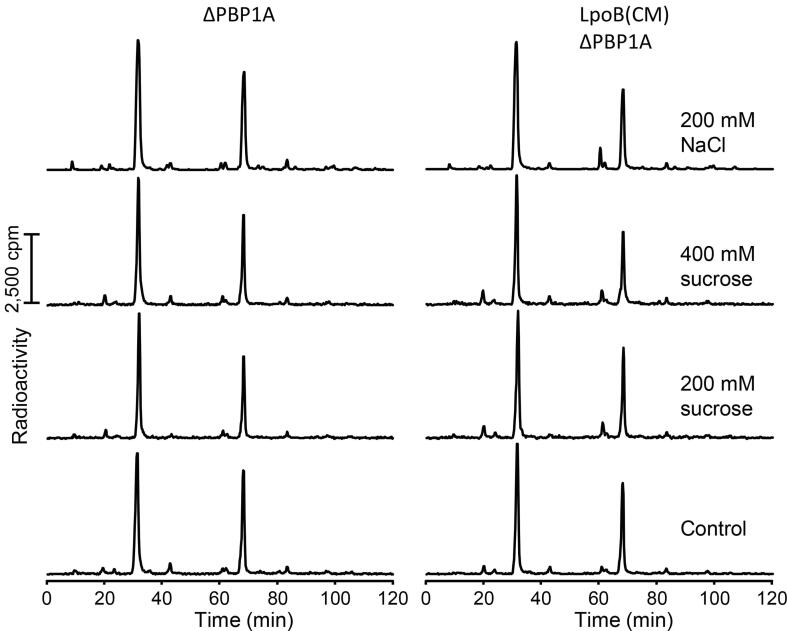
Composition of new PG synthesized after an osmotic upshift in ΔPBP1A and LpoB(CM) ΔPBP1A cells. Muropeptides were prepared from cells grown in LB medium with no salt or LB medium supplemented with NaCl or sucrose at indicated concentrations. Muropeptides were analyzed using HPLC. Muropeptide peaks are quantified in Supplemental Tables 2 and 3.

We next sought to examine the effects of mis-localizing LpoB on the composition of PG under osmotic upshift. We grew strains in LB medium with no salt and added 200 mM NaCl at an optical density (OD) of 0.4 followed by growth for further 30 min. Control cells did not receive NaCl. Cells were harvested, their PG was isolated and digested with the muramidase cellosyl, followed by the analysis of the muropeptide composition by high-performance liquid chromatography (HPLC). We found that the LpoB(CM) ΔPBP1A cells had slightly reduced cross-linkage and increased average glycan chain length compared to ΔPBP1A cells, but overall the PG composition looked similar (Supplemental Fig. 1, Supplemental Table 1). We next wanted to determine the composition of the PG produced during the osmotic upshift. For this, we repeated the experiment but this time added [^3^H]Glc*N*Ac 5 min before the osmotic upshift and present during the 30 min growth period after the osmotic upshift with 200 mM NaCl, 200 mM sucrose or 400 mM sucrose. Muropeptides were prepared as before and analyzed by HPLC connected to a radioactivity flowthrough detector. We observed that the composition of the PG produced during and after the osmotic upshift was similar in both strains and in the different upshift experiments (Fig. 3, Supplemental Tables 2 and 3). Altogether our data indicate that mis-localization of LpoB does not substantially affect the composition of the PG.

### Mis-localization of LpoB affects PG synthesis rate after an osmotic upshift

It was hypothesized that OM anchored LpoA and LpoB adjust PG synthesis by their cognate PBPs in response to the overall cellular growth rate via the size of pores in the PG (Typas et al., 2012). The PG layer is flexible and can stretch or shrink when it is exposed to low or high media osmolality conditions. At low external osmolality, when the PG is more stretched and pore sizes are larger, Lpo proteins can traverse the PG layer easier and more efficiently activate their cognate synthases and therefore PG synthesis. By contrast, in high external osmolality conditions, when the pores are smaller due to a shrunken PG layer, the access of Lpo proteins to their synthases is more restricted and therefore PG synthesis rate is reduced. Therefore, we hypothesized that with CM-tethered LpoB the activation of PBP1B should be less responsive to osmolality conditions and PG synthesis should remain high if cells are transferred into high osmolality medium.

**Fig. 4:**
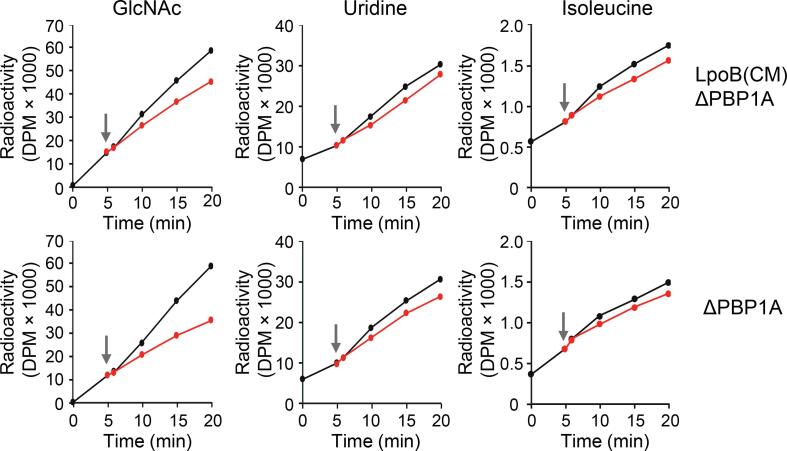
Metabolic labelling of PG, DNA, and protein synthesis in ΔPBP1A and LpoB(CM) ΔPBP1A cells upon osmotic upshift. ΔPBP1A and LpoB(CM) ΔPBP1A cells were grown in LB medium with no salt until the OD_578_ of ∼ 0.3 and [^3^H]Glc*N*Ac or [^3^H]isoleucine or [^3^H]uridine were added to the culture. After 5 min, each culture was divided into two parts. One part was supplemented with 200 mM (grey arrow)and the second part received the same amount of LB media with no NaCl. Sample aliquots were collected for 20 min in 5 min time intervals and the cellular radioactivity was measured as described in Methods. The red circles represent the incorporation of radioactivity under osmotic upshift conditions, the black circles represent incorporation of radioactivity in the control sample (no NaCl added). (For interpretation of the references to colour in this figure legend, the reader is referred to the web version of this article.)

To test this hypothesis, we measured the rate of incorporation of radiolabeled [^3^H]Glc*N*Ac into PG in LpoB(CM) ΔPBP1A cells compared to ΔPBP1A cells with OM-anchored LpoB with or without an osmotic upshift with 0.2 M sucrose (Fig. 4). The osmotic shock caused a 39.8 % reduction in the incorporation of [^3^H]Glc*N*Ac into PG in ΔPBP1A cells, but only a 22.7 % reduction in [^3^H]Glc*N*Ac incorporation in LpoB(CM) ΔPBP1A cells. This result indicates that LpoB(CM) ΔPBP1A cells down-regulate PG synthesis rate less in response to an osmotic upshift (Fig. 4). As expected, the mis-localized LpoB affected only PG synthesis and not the synthesis of nucleic acids or proteins, as the incorporation rates of radioactive [^3^H]uridine or [^3^H]isoleucine were less affected by the osmotic upshift indicating a slower response of other cellular processes compared to PG synthesis and, more importantly, [^3^H]uridine and [^3^H]isoleucine were incorporated at similar rates in LpoB(CM) ΔPBP1A and ΔPBP1A cells upon the osmotic upshift. This indicates that the mis-localization of LpoB to the CM specifically affects the synthesis rate of PG in response to an osmotic upshift.

**Fig. 5:**
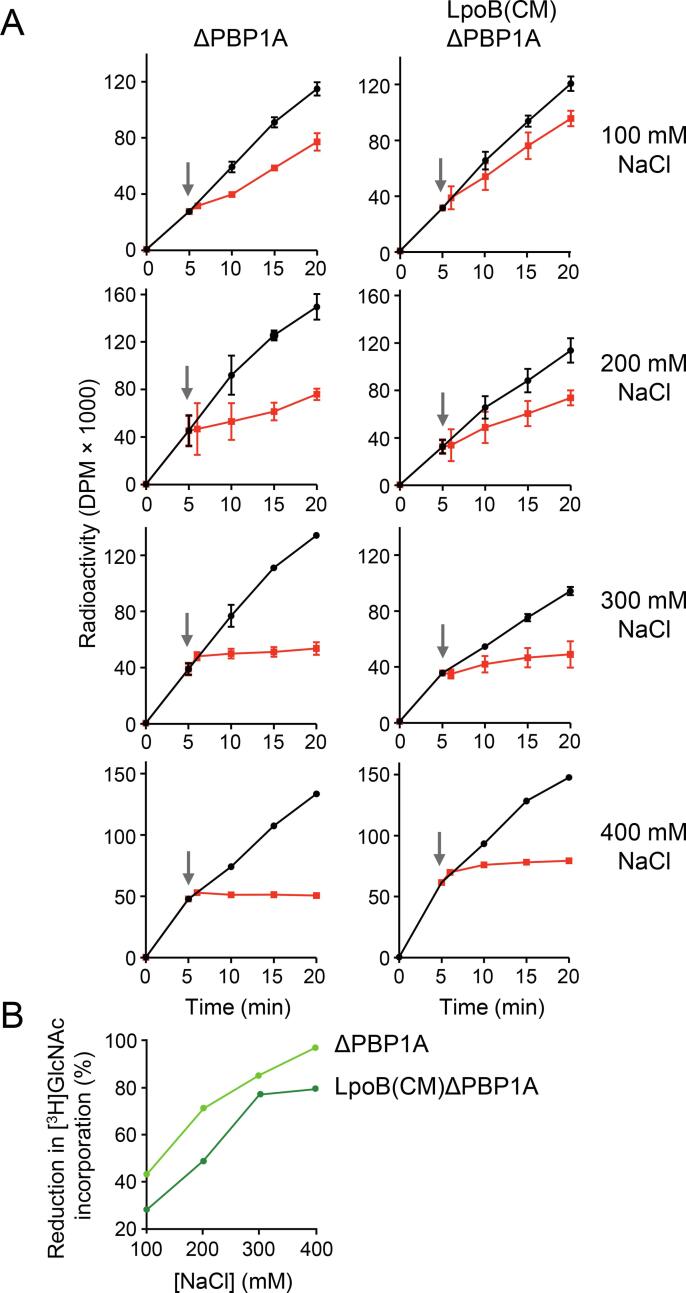
Metabolic labelling of PG in ΔPBP1A and LpoB(CM) ΔPBP1A cells upon osmotic upshift. (**A**) The [^3^H]Glc*N*Ac incorporation experiment was done as in Fig. 4 but with different concentrations of NaCl for osmotic upshift. Values are mean ± variation of two independent experiments (100, 200 and 300 mM NaCl). The experiment with 400 mM NaCl was performed once. (B) Summary of the results shown as % reduction in [^3^H]Glc*N*Ac incorporation depending on the extent of osmotic upshift.

To determine the effect of an osmotic upshift on the PG biosynthesis rate in more detail, we repeated the measurement of [^3^H]Glc*N*Ac incorporation into PG upon a range of osmotic upshifts, by varying the concentration of added NaCl from 100 mM to 400 mM (Fig. 5A). After a low osmotic shock with 100 mM NaCl, LpoB(CM) ΔPBP1A displayed a 28 % reduction compared to no salt condition, whereas ΔPBP1A cells showed a 43 % reduction in the incorporation rate (Fig. 5B). As may be expected, by increasing the NaCl concentration we observed a greater reduction in the rate of [^3^H]Glc*N*Ac incorporation in both strains. At 200 mM salt, LpoB(CM) ΔPBP1A and ΔPBP1A showed a 49 % and 71 % reduction, respectively, in the rate of [^3^H]Glc*N*Ac incorporation (Fig. 5). An osmotic shock generated by adding 300 mM NaCl resulted in 77 % and 85 % reduction in the rate of [^3^H]Glc*N*Ac incorporation into LpoB(CM) ΔPBP1A and ΔPBP1A strains, respectively. Ultimately, 400 mM NaCl osmotic shock almost completely inhibited incorporation of [^3^H]Glc*N*Ac in ΔPBP1A, while LpoB(CM) ΔPBP1A displayed 79 % reduction. The reduction in [^3^H]Glc*N*Ac incorporation into LpoB(CM) ΔPBP1A and ΔPBP1A strains in 100–400 mM osmotic upshift has been summarized as percentage in Fig. 5B. Altogether, these data suggest that the OM localization of LpoB is not the sole determinant for reducing PG synthesis rate after an osmotic upshift but it contributes to a significant extent (about 30–80 %) to the cells ability to down-regulate the PG synthesis.

## Discussion

PG synthesis is regulated by cytoskeletal elements and lipoproteins from cytoplasmic membrane and OM, respectively (Typas et al., 2010). In *E. coli* two OM lipoproteins, LpoA and LpoB, activate two major PG synthases, PBP1A and PBP1B, respectively (Typas et al., 2010, Paradis-Bleau et al., 2010, Egan et al., 2014, Egan et al., 2018). Each lipoprotein is essential for the function of its cognate PG synthase, while each pair can compensate the function of the other pair in non-stress conditions. Under certain stress conditions, the cells need PBP1B/LpoB to survive (Morè et al., 2019, de Pedro et al., 2001). One of the key unanswered questions is to what extent the function of the Lpo lipoproteins is dependent on their localization to the OM. In the present study we investigated the effects of mislocalization of LpoB on PG synthesis in *E. coli*.

### Mis-localized LpoB is able to activate PBP1B

It has been shown that LpoA-PBP1A and LpoB-PBP1B have overlapping functions and therefore the presence of only one of them at each time is essential for *E. coli* cells survival (Typas et al., 2010). Generating a strain lacking PBP1A (ΔPBP1A) renders PBP1B essential. ΔPBP1A cells grew both in LB media and on LB plates supplemented with low to high NaCl concentrations restating the fact that PBP1B suffices for cell growth and survival. Changing the LpoB localization from OM to CM in ΔPBP1A cells [LpoB(CM) ΔPBP1A] (by changing the LpoB sorting signal of the chromosomal copy of *lpoB* (Typas et al., 2010) enabled us to investigate the significance of its OM localization on PG synthesis (Fig. 1). Similar to ΔPBP1A cells, LpoB(CM) ΔPBP1A cells were able to grow in LB no/low NaCl conditions, however, they failed to grow at high NaCl conditions (Fig. 2A-B) consistent with previous work (Typas et al., 2010). These results suggest that mislocalized LpoB is still able to bind and activate PBP1B, although it is not fully functional at high osmolarity conditions which leads to lysis. The lack of LpoB function at high NaCl condition could be caused by a poorer activation of PBP1B due to reduced binding affinity in the presence of high NaCl concentration or the less efficient interaction with PBP1B through the pores of a more relaxed PG, or both.

### Mis-localization of LpoB impacts cells morphology

PG synthesis rate is adjusted to overall cellular growth, i.e., when the osmolarity is low or cells grow faster, the PG synthesis rate is higher and, in contrast, when the osmolarity is high or cells grow slower, the PG synthesis rate drops. Therefore, the increased length and irregularity of cells with mis-localized LpoB is consistent with a failure to downregulate PG synthesis or a change in the composition of muropeptides. Analysis of muropeptides composition showed similar PG composition upon tethering LpoB to the CM (Fig. 3 and Fig. S1, Tables S1 and S2). Strikingly, the PG sacculi prepared from these cells are longer and display irregularity in many directions, further affirming that mis-localization of LpoB impacts the PG synthesis level not its composition.

### Mis-localized LpoB is impaired in downregulating PG synthesis

The tertiary structure of LpoB (Egan et al., 2014) has paved the way to better understand how this lipoprotein located on OM regulate the PG synthesis. LpoB and presumably LpoA have elongated conformations that are about 145 Å long, which enables them to traverse the periplasm to reach their cognate PG synthases. PBP1B has a small noncatalytic domain (UB2H) as a docking site for LpoB binding that resides in the space between the CM and PG layer (Egan et al., 2014, Sung et al., 2009). Therefore, hindering the passage of the lipoprotein activators through PG layer can potentially prevent PG synthase activation. It is also known that PG possesses an elastic structure (Vollmer and Höltje, 2004), which can expand or shrink in response to turgor pressure (Cayley et al., 2000). Based on these information, Typas and colleagues proposed a regulatory mechanism in which the OM localization of LpoB and PG pore size affect the activation of the key class A PBP PG synthases (Typas et al., 2012, Typas et al., 2010). Results from our incorporation assays are consistent with this hypothesis. While ΔPBP1A cells substantially lowered the rate of PG synthesis upon shift to high NaCl conditions, LpoB(CM) ΔPBP1A cells continued to incorporate additional [^3^H]Glc*N*Ac into PG (Fig. 5). This suggests that since LpoB(CM) no longer needs to traverse through PG layer, it was able to better activate PBP1B and consequently the cell’s overall PG synthesis was higher than normal. Presumably, other effects contributed to a recognizable reduction in PG rate even in the LpoB(CM) ΔPBP1A cells upon osmotic upshift. First, the high NaCl concentration entering the periplasm could weaken the affinity between PBP1B and LpoB. Second, the mis-regulation of PG synthesis caused by LpoB(CM) likely affects only PBP1B but not PG synthesis mediated by SEDS-class B complexes, hence the activity of the latter is likely still responsive and downregulated upon the osmotic upshift. Members of the elongasome (MreCD) and divisome (FtsQLB complex, FtsN) have been implicated in regulating PG synthesis (Typas et al., 2012, Egan et al., 2020) but little is known about how the elongasome and divisome respond to an osmotic shift. It is also possible that other proteins contribute in cells ability to downregulate the PG synthesis rate, which still need to be discovered.

## Conclusion

Our results supports the model that outer-membrane anchored lipoproteins, such as LpoB, can regulate PG synthesis rate by spanning the periplasm and reaching their cognate PG synthases, such as PBP1B.

## Materials and methods

### Bacterial strains and growth media

Strains BW25113ΔPBP1A and BW25113 LpoB(CM) ΔPBP1A used in this study were described previously (16). Unless otherwise noted cells were grown at 37 °C in LB medium without NaCl. and if necessary kanamycin (30 or 50 mg/L) was included in overnight pre-cultures but not in the culture when assays were performed. NaCl and sucrose were prepared as concentrated stocks, filter sterilised using 0.22 µm syringe filters and added to the required concentrations as noted.

### Bacterial growth curves and spot plate assay

ΔPBP1A and LpoB(CM)ΔPBP1A cells were streaked on LB-Kanamycin plates (30 μg/ml) to obtain single colonies. Single colonies were used to prepare overnight cultures in LB medium supplemented with 30 mg/L kanamycin. LB-kanamycin media was inoculated using the overnight cultures and cells were grown to an OD_578_ of 0.3 at 37 °C. At this stage each culture was serially diluted and spotted on LB agar plates containing different concentrations of NaCl (from 0 mM to 600 mM), or osmotically shocked by adding 400 or 600 mM NaCl. The growth of each culture was monitored by taking the OD_578_ for 4 h in 30 min time intervals. LB agar plates were incubated overnight at 37 °C.

### Election microscopy of PG sacculi

ΔPBP1A and LpoB(CM) ΔPBP1A cells were transferred into LB media (no salt or containing 600 mM NaCl) an incubated at 37 °C to an OD_578_ of 0.5–0.6. At this stage, live cells were used for direct visualization by light microscopy or sacculi were purified for imaging by electron microscopy (de Pedro et al., 1997). To prepare sacculi ΔPBP1A and LpoB(CM) ΔPBP1A cells were harvested by centrifuging at 10,000 × *g* for 15 min at 4 °C. The cell pellet was resuspended in ice-cold NaCl solution (0.9 g/L). Resuspended cells were boiled at 8 % (w/v) sodium dodecyl sulfate (SDS) for 30 min. SDS-boiled samples were transferred into a capped glass tube and incubated overnight at 80 °C in a water bath. The next day, cell pellets were collected by centrifugation at 400,000 × *g* for 30 min at 30 °C. The cell pellet was resuspended in 2.5 ml of 4 % (w/v) SDS and incubated for 4 h in a boiling water bath. The sample was centrifuged as before and cell pellet was washed in 1 % (w/v) SDS and further incubated at 100 °C for 4 h or overnight at 80 °C. The sample was centrifuged as before and the cell pellet was resuspended in 50 mM sodium phosphate (pH 7.4) supplemented with α-chymotrypsin (100 μg/ml), and incubated at 37 °C for 4 h. At the end of incubation time, the same amount of α-chymotrypsin was added and samples were further incubated overnight at 37 °C. Next day 1 % (w/v) SDS was added to each sample, and samples were incubated at 100 °C for 4 h. The sample was centrifuged as before and the cell pellet was resuspended in 1 % (w/v) SDS, and incubated for 2 h in a boiling water bath. The sample was centrifuged as before and the cell pellet was resuspended in 100 μl of Milli-Q water and supplemented with 0.1 % (w/v) sodium azide. Samples were stored at 4 °C for same day use or at −20 °C for longer term.

Carbon-pioloform-coated copper grids were glow discharged (10 min) and floated for 15 min on drops of sacculus suspensions. The excess amount of liquid was removed using filter papers, and grids were air dried for 10 min. Grids were washed three times by floating on Milli-Q water drops and stained by floating on 1 % (w/v) uranyl acetate solution for 1 min. Grids were washed in Milli-Q water, air dried at room temperature and imaged by transmission electron microscopy.

### PG isolation and muropeptide analysis

ΔPBP1A and LpoB(CM) ΔPBP1A cells were grown at 37 °C in LB (no NaCl) to an OD_600_ of 0.4. Each culture was divided into two and sterile NaCl equivalent to 200 mM was added to one of the aliquots. The other culture received an equivalent volume of LB (no NaCl). Both cultures were incubated for further 30 min. The cells were harvested by centrifugation at 4,000 × *g* and cells were resuspended in phosphate buffered saline (PBS). The cells were then added to boiling 8 % (w/v) SDS and the PG was prepared and analyzed as previously published (Glauner, 1988).

### Metabolic labelling of PG, DNA, and proteins

The rates of PG, protein and RNA synthesis in ΔPBP1A and LpoB(CM) ΔPBP1A cells were simultaneously determined under different osmotic shock conditions by measuring the incorporation rates of [^3^H]Glc*N*Ac into peptidoglycan (Kraft et al., 1999); [ ^3^H]isoleucine into proteins and [^3^H]uridine into RNA (Henry et al., 1992), respectively. Exponentially growing cells in a 40 ml culture with an OD_578_ of ∼0.3 were labelled with either [^3^H]Glc*N*Ac (37 kBq/ml; 10 µg/ml Glc*N*Ac), [^3^H]isoleucine (37 kBq/ml, 1 mM) or [^3^H]uridine (37 kBq/ml, 1 mM) in LB medium without NaCl at 37 °C. 0.5 ml aliquots of each culture were taken immediately after the addition of radioisotope and in 5 min time intervals for another 20 min. 5 min after the addition of radioisotope, the culture was split into two, with one aliquot receiving NaCl or sucrose (to different final concentration of 100 mM to 400 mM). The control culture received an equivalent amount of LB media with no NaCl. At each time point, 0.5 ml of each culture was collected and added to either 3 ml of boiling 8 % (w/v) SDS (for [^3^H]Glc*N*Ac labelled sample) or 3 ml of ice-cold 20 % (w/v) trichloroacetic acid (TCA). [^3^H]Glc*N*Ac labelled samples were boiled at 100 °C for 30 min, [^3^H]isoleucine and [^3^H]uridine samples were incubated on ice for 30 min. The boiled [^3^H]Glc*N*Ac labelled samples were filtered through a 0.22 µm GS cellulose nitrate filter (Millipore), washed three times with 3 ml of 0.1 M LiCl and three times with 3 ml of Milli-Q water. The TCA precipitates of [^3^H]isoleucine and [^3^H]uridine samples were filtered through a 0.22 µm Whatman GF/C filters and washed 3 times with 3 ml of ice-cold 10 % (w/v) TCA and 3 times with 3 ml of ice-cold 95 % (w/v) ethanol with 0.1 M HCl. All filters were transferred into a scintillation vial, dried and the radioactivity on the dried filters was measured in the presence of 5 ml scintillation cocktail (Ecoscint A, National Diagnostics) in a HIDEX 300SL scintillation counter.

### CRediT authorship contribution statement

**Ali A. Kermani:** Conceptualization, Investigation, Validation, Writing – original draft, Writing – review & editing, Visualization. **Jacob Biboy:** Conceptualization, Investigation, Validation, Writing – original draft, Writing – review & editing, Visualization. **Daniela Vollmer:** Investigation. **Waldemar Vollmer:** Conceptualization, Methodology, Validation, Data curation, Writing – original draft, Writing – review & editing, Supervision, Project administration, Funding acquisition.

## Declaration of Competing Interest

The authors declare that they have no known competing financial interests or personal relationships that could have appeared to influence the work reported in this paper.
