# Supplementary material for: Outer membrane-anchoring enables LpoB to regulate peptidoglycan synthesis rate
Source: Cell Surf. 2022 Oct 20;8:100086. doi: 10.1016/j.tcsw.2022.100086 (PMC9593243; doi:10.1016/j.tcsw.2022.100086)
Supplement: Supplementary data 1 [file mmc1.pdf]

Supplementary Information

Outer membrane-anchoring enables LpoB to regulate peptidoglycan synthesis rate

Ali A. Kermani[1^#*], Jacob Biboy[1#], Daniela Vollmer[1] and Waldemar Vollmer[1*]

[1] Centre for Bacterial Cell Biology, Biosciences Institute, Newcastle University, Richardson Road, Newcastle upon Tyne, NE2 4AX, UK.

^ Present address: Department of Structural Biology, St. Jude Children's Research Hospital, Memphis, TN, 38105, United States

[#] Contributed equally.

* Corresponding authors: Ali A. Kermani, Email: akermani@stjude.org; Waldemar Vollmer, Email: w.vollmer@ncl.ac.uk

Supplementary Information

Supplementary Table 1: Muropeptide composition of strains after osmotic upshift.

Supplementary Table 2: Muropeptide composition of newly synthesized PG after osmotic upshift with NaCl.

Supplementary Table 3: Muropeptide composition of newly synthesized PG after osmotic upshift with sucrose.

Supplementary Figure 1: PG composition of ΔPBP1A and LpoB(CM)ΔPBP1A cells.

Supplementary Table 1. Muropeptide composition of strains after osmotic upshift.

Muropeptide or feature[1]	Relative peak area (%)				
	BW25113[2]	ΔPBP1A		LpoB(CM) ΔPBP1A	
		no NaCl	0.2 M NaCl	no NaCl	0.2 M NaCl
Tri	5.3	7.0	5.3	6.2	4.5
TetraGly[4]	1.4	2.2	1.7	3.2	2.5
Tetra	40.9	38.1	38.5	42.7	42.7
Di	1.3	1.9	1.3	1.3	0.7
Tri-LysArg	3.3	3.0	3.8	2.4	2.7
TetraTri(Dap)	2.0	1.6	2.9	4.2	9.5
TetraTetraGly[4]	1.7	1.8	1.8	2.2	1.7
TetraTri	1.8	1.5	1.4	1.8	1.3
TetraTetra	33.4	31.0	31.2	28.3	27.0
TetraPenta	1.0	0.7	0.8	0.5	0.6
TetraTetraTri	1.7	0.9	1.4	0.5	0.7
TetraTri-LysArg	0.0	2.6	2.3	1.5	1.2
TetraTetraTetra	3.1	2.4	2.6	1.6	1.8
TetraTriAnh	0.0	2.6	1.6	1.2	0.6
TeraTetraAnh I	1.3	0.7	0.9	0.7	0.9
TeraTetraAnh II	0.9	1.0	1.0	1.0	0.8
TetraTetraTetraAnh	0.9	1.3	1.5	0.6	0.7
Monomers (total)	52.3	52.1	50.7	55.9	53.2
Dimers (total)	42.0	43.3	43.8	41.4	43.6
Trimers (total)	5.7	4.6	5.5	2.8	3.1
Dipeptides (total)	1.3	1.9	1.3	1.3	0.7
Tripeptides (total)	7.1	8.2	6.8	7.4	5.5
Tetrapeptides (total)	82.8	79.6	80.3	83.0	82.6
Pentapeptides (total)	0.5	0.3	0.4	0.2	0.3
Chain ends (%)	1.4	1.3	1.5	1.1	1.1
Av. glycan length (DS)[2]	72	77	68	93	93
LD-crosslinks (%)	1.00	0.79	1.46	2.09	4.75
DD-crosslinks (%)	23.8	23.9	24.1	20.5	19.2
Degree of cross-linkage	24.8	24.7	25.6	22.5	23.9
Peptides in cross-links	47.7	47.9	49.3	44.1	46.8

Muropeptide names according to Glauner, 1988.

[2] The parental strain BW25113 was grown in standard LB medium.

[3] Average glycan chain length in disaccharide (DS) units was calculated from the percentage of anhydroMur*N*Ac containing muropeptides.

Supplementary Table 2. Muropeptide composition of newly synthesized PG after osmotic upshift with NaCl.

Muropeptide or feature[1]	Relative peak area (%)[2]			
	ΔPBP1A		LpoB(CM) ΔPBP1A	
	no NaCl	0.2 M NaCl	no NaCl	0.2 M NaCl
Tri	2.2 ± 0.6	2.0 ± 1.4	2.9 ± 0.5	1.1 ± 0.5
TetraGly[4]	1.3 ± 0.3	1.3 ± 0.0	1.9 ± 0.1	1.3 ± 0.5
Tetra	51.4 ± 2.6	50.8 ±	53.7 ±	40.8 ± 17.7
Di	1.3 ± 1.4	1.7 ± 1.3	1.4 ± 0.3	1.4 ± 0.7
Penta	3.1 ± 1.2	2.6 ± 1.1	2.4 ± 0.6	3.6 ± 2.5
TetraTri(Dap)	1.7 ± 0.5	1.7 ± 0.8	3.5 ± 0.9	4.4 ± 0.6
TetraTetraGly[4]	n.d.	0.3 ± 0.6	n.d.	0.5 ±0.8
TetraTri	2.0 ± 0.2	1.9 ± 0.6	3.2 ± 0.6	0.9 ± 1.0
TetraTetra	30.3 ± 2.8	31.2 ± 8.7	26.6 ±	39.3 ±13.4
TetraPenta	1.4 ± 1.5	1.2 ± 0.3	0.6 ± 0.6	2.0 ± 1.3
TetraTetraTri	0.3 ± 0.5	n.d.	n.d.	0.6 ± 1.0
TetraTetraTetra	2.6 ± 0.5	2.4 ± 0.5	1.7 ±0.1	2.1 ± 1.2
TetraTriAnh	0.2 ± 0.4	0.2 ± 0.4	0.3 ± 0.4	0.4 ± 0.7
TeraTetraAnh I	0.5 ± 0.4	1.0 ± 0.3	1.1 ± 0.4	1.0 ± 0.1
TeraTetraAnh II	0.6 ± 0.5	0.5 ± 0.5	0.7 ± 0.6	0.6 ± 0.5
TetraTetraTri Anh	n.d.	n.d.	n.d.	n.d.
TetraTetraTetra Anh	1.2 ± 1.3	1.2 ± 1.2	0.2 ± 0.4	0.3 ± 0.5
Monomers (total)	59.3 ± 2.6	58.4 ± 9.7	62.2 ±	48.1 ± 14.1
Dimers (total)	36.6 ± 2.6	38.0 ±8.0	35.9 ±	48.9 ± 12.4
Trimers (total)	4.1 ± 1.5	3.6 ±1.7	1.9 ±0.4	2.9 ± 2.1
Dipeptides (total)	1.3 ± 1.4	1.7 ± 1.3	1.4 ± 0.3	1.4 ± 0.7
Tripeptides (total)	4.2 ± 0.4	3.9 ± 1.0	6.3 ± 0.3	4.1 ± 0.1
Tetrapeptides (total)	89.9 ±1.3	90.7 ±2.8	88.3 ±	88.1 ± 3.3
Pentapeptides (total)	2.4 ± 1.2	3.2 ± 1.0	2.7 ± 0.9	1.0 ± 0.6
Chain ends (anhydro)	1.0 ± 0.5	1.3 ± 0.4	1.1 ± 0.5	1.1 ± 0.5
Av. glycan length (DS)[2]	112 ± 49	87 ± 35	106 ± 48	109 ± 62
LD crosslinks	0.8 ± 0.3	0.9 ± 0.4	1.7 ± 0.5	2.2 ± 0.3
DD crosslinks	20.2 ± 1.3	20.4 ± 5.3	17.5 ±	24.2 ± 7.6
Degree of cross-linkage	21.0 ± 1.4	21.4 ± 5.1	19.2 ±	26.4 ± 7.3
% Peptides in cross-links	40.7 ± 2.6	41.6 ± 9.7	37.8 ±	51.9 ± 14.1

[1] Muropeptide names according to Glauner, 1988.

[2] Values are mean ± SD of 3 PG preparations, n.d., not detected.

[3] Average glycan chain length in disaccharide (DS) units was calculated from the percentage of anhydroMur*N*Ac containing muropeptides.

Supplementary Table 3. Muropeptide composition of newly synthesized PG after osmotic upshift with sucrose.

Muropeptide[1] or feature	Relative peak area (%)[2]					
	ΔPBP1A			LpoB(CM) ΔPBP1A		
	no sucrose	0.2 M sucrose	0.4 M sucrose	no sucrose	0.2 M sucrose	0.4 M sucrose
Tri	2.4 ± 0.5	3.1 ± 0.3	3.0 ± 0.3	2.2 ± 0.5	4.2 ± 1.0	5.0 ± 0.2
TetraGly[4]	1.3 ± 0.1	1.3 ± 0.1	1.3 ± 0.4	1.7 ± 0.3	1.8 ± 0.3	1.9 ± 0.5
Tetra	48.6 ± 4.0	51.8 ± 4.2	50.6 ± 2.6	53.1 ± 3.3	48.5 ± 1.9	48.1 ± 0.8
Di	1.3 ± 0.2	1.1 ± 0.3	0.3 ± 0.3	0.8 ± 0.4	0.6 ± 0.1	0.7 ± 1.2
Penta	2.4 ± 1.6	1.1 ± 0.2	3.0 ± 0.2	1.8 ± 1.0	1.0 ± 0.9	3.6 ± 0.3
TetraTri(Dap)	1.7 ± 1.0	2.0 ± 1.0	2.9 ± 1.0	2.2 ± 0.7	3.7 ± 1.3	5.4 ± 1.0
TetraTetraGly[4]	n.d.	0.3 ± 0.5	0.1 ± 0.3	n.d.	0.6 ± 0.5	0.8 ± 0.8
TetraTri	1.5 ± 0.8	1.5 ± 0.7	1.9 ± 0.5	2.1 ± 0.4	1.3 ± 0.8	2.0 ± 0.7
TetraTetra	34.7 ± 1.8	32.9 ± 2.8	30.7 ± 1.3	31.8 ± 2.9	33.3 ± 3.2	26.1 ± 3.7
TetraPenta	0.5 ± 0.5	0.6 ± 0.6	0.9 ± 0.4	0.6 ± 0.4	0.4 ± 0.3	0.9 ± 0.3
TetraTetraTri	0.7 ± 0.2	0.2 ± 0.4	0.7 ± 0.6	0.4 ± 0.3	0.4 ± 0.3	0.5 ± 0.5
TetraTetraTetra	2.2 ± 0.9	2.1 ± 0.1	1.8 ± 0.7	1.5 ± 0.5	1.9 ± 0.1	1.4 ± 0.4
TetraTriAnh I	0.7 ± 1.1	0.4 ± 0.4	0.7 ± 1.1	0.3 ± 0.5	0.2 ± 0.3	0.8 ± 0.5
TeraTetraAnh I	0.7 ± 0.2	1.1 ± 0.4	0.6 ± 0.6	0.6 ± 0.3	1.2 ± 0.2	0.9 ± 0.3
TeraTetraAnh II	0.7 ± 0.4	0.4 ± 0.4	0.6 ± 0.5	0.6 ± 0.3	0.5 ± 0.5	1.2 ± 0.6
TeraTetraTri Anh	0.2 ± 0.4	n.d.	0.2 ± 0.4	n.d.	n.d.	0.3 ± 0.4
TetraTetraTetra Anh	0.3 ± 0.3	0.2 ± 0.3	0.6 ±0.6	0.3 ± 0.4	0.4 ± 0.4	0.5 ± 0.9
Monomers (total)	56.0 ± 2.2	58.3 ± 4.3	58.3 ± 2.6	59.5 ± 3.1	56.1 ± 3.1	59.2 ± 1.8
Dimers (total)	40.6 ± 2.3	39.3 ± 3.9	38.4 ± 2.1	38.2 ± 3.0	41.3 ± 2.4	38.1 ± 2.4
Trimers (Total)	3.4 ± 0.7	2.5 ± 0.4	3.3 ± 0.6	2.3 ± 0.4	2.6 ± 0.8	2.7 ± 1.1
Dipeptides (total)	1.3 ± 0.2	1.1 ± 0.3	0.3 ± 0.3	0.8 ± 0.4	0.6 ± 0.1	0.7 ± 1.2
Tripeptides (total)	4.7 ± 1.4	5.1 ± 0.5	6.0 ± 0.8	4.6 ± 1.1	6.9 ± 1.6	9.3 ± 0.8
Tetrapeptides (total)	90.5 ± 3.8	91.7 ± 1.0	88.7 ± 1.7	91.5 ± 2.8	89.9 ± 2.8	83.3 ± 2.2
Pentapeptides (total)	2.7 ± 1.8	1.4 ± 0.3	3.5 ± 0.4	2.1 ± 1.2	1.2 ± 0.8	4.0 ± 0.2
Chain ends (anhydro)	1.2 ± 0.5	1.0 ± 0.3	1.2 ± 0.4	0.9 ± 0.1	1.1 ± 0.3	1.7 ± 0.4
Av. glycan length (DS)[3]	91 ± 30	104 ± 36	92 ± 39	117 ± 13	97 ± 33	60 ± 16
LD crosslinks	0.8 ± 0.5	1.0 ± 0.5	1.4 ± 0.5	1.1 ± 0.4	1.9 ± 0.7	2.7 ± 0.5
DD crosslinks	21.8 ± 0.8	20.3 ± 2.0	20.0 ± 0.9	19.5 ± 1.4	20.5 ± 2.3	18.2 ± 1.3
Degree of cross-linkage	22.6 ± 1.1	21.3 ± 2.2	21.4 ± 1.4	20.6 ± 1.6	22.4 ± 1.6	20.9 ± 0.8
% Peptides in cross-links	44.0 ± 2.2	41.7 ± 4.3	41.7 ± 2.6	40.5 ± 3.1	43.9 ± 3.1	40.8 ± 1.8

[1] Muropeptide names according to Glauner, 1988.

[2] Values are mean ± SD of 3 PG preparations; n.d., not detected.

[3] Average glycan chain length in disaccharide (DS) units was calculated from the percentage of anhydroMur*N*Ac containing muropeptides.

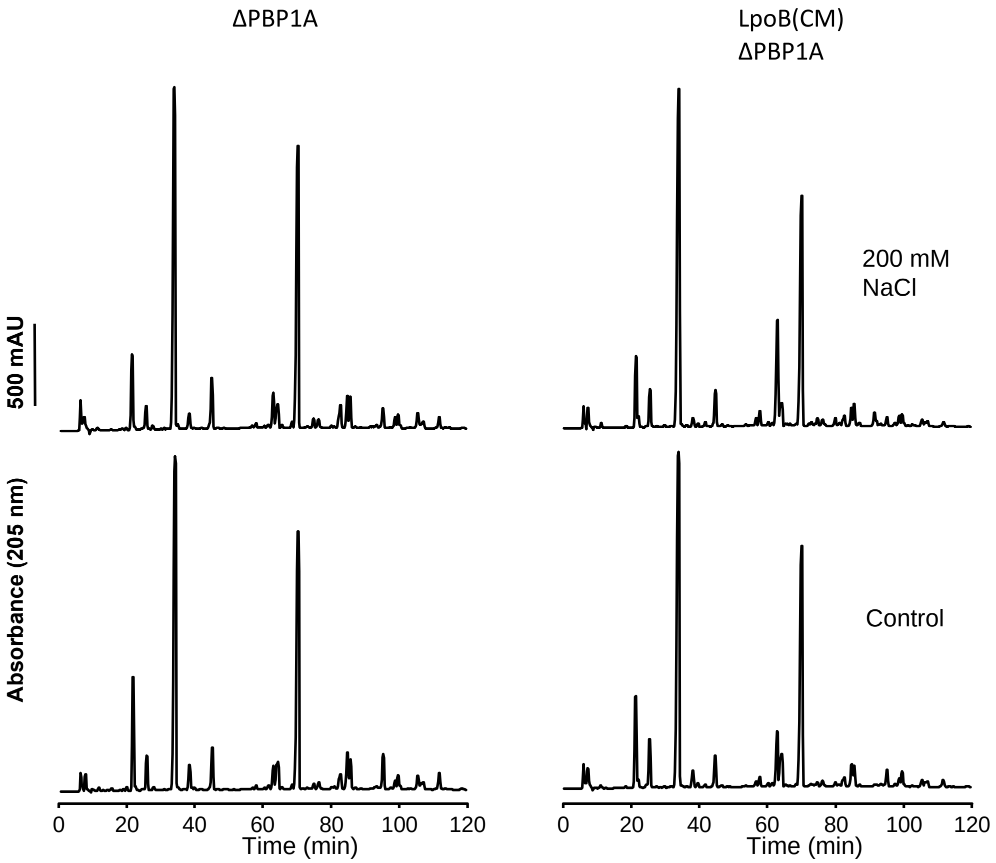

Figure S1. PG composition of ΔPBP1A and LpoB(CM)ΔPBP1A cells. Muropeptides prepared from cells grown in LB medium with no salt or supplemented 200 mM NaCl. Muropeptides were analyzed using HPLC. Related to Supplemental Table 1.
